# Cytotoxicity and Efficacy in Debris and Smear Layer Removal of HOCl-Based Irrigating Solution: An In Vitro Study

**DOI:** 10.3390/jfb13030095

**Published:** 2022-07-15

**Authors:** Goda Bilvinaite, Ruta Zongolaviciute, Saulius Drukteinis, Virginija Bukelskiene, Elisabetta Cotti

**Affiliations:** 1 Institute of Dentistry, Faculty of Medicine, Vilnius University, Zalgirio 115, LT-08217 Vilnius, Lithuania; goda.bilvinaite@gmail.com (G.B.); ruta757@gmail.com (R.Z.); 2 Department of Biological Models, Life Sciences Center, Institute of Biochemistry, Vilnius University, LT-10257 Vilnius, Lithuania; virginija.bukelskiene@bchi.vu.lt; 3 Department of Conservative Dentistry and Endodontics, University of Cagliari, 09124 Cagliari, Italy; cottiendo@gmail.com

**Keywords:** cytotoxicity, debris, human gingival fibroblasts, irrigation, root canal, smear layer, Sterilox, super-oxidized water

## Abstract

In the present study we evaluated the cytotoxicity of super-oxidized water on human gingival fibroblasts and its efficacy in debris and smear layer removal from root canal walls. Cultured gingival fibroblasts were exposed to super-oxidized water (Sterilox), which was diluted in Iscove’s modified Dulbecco’s medium (IMDM) at 30%, 40%, 50%, 60% and 70% concentrations. The control group was maintained in IMDM. The cell viability was evaluated by means of an MTT assay after incubation periods of 1 h, 2 h, 24 h and 48 h. Pathological cellular changes were also observed under fluorescence and phase contrast microscopes. The efficacy in debris and smear layer removal was evaluated in comparison to the conventional application of sodium hypochlorite (NaOCl) and ethylenediaminetetraacetic acid (EDTA). Forty maxillary premolars were randomly divided into two equal groups (*n* = 20) and shaped with ProTaper NEXT rotary instruments using Sterilox or NaOCl/EDTA for irrigation. Afterwards, roots were split longitudinally and examined under a scanning electron microscope. The results revealed that super-oxidized water and sterile distilled water have acceptable biological properties for endodontic applications at concentrations up to 50% (*p* > 0.05). Moreover, super-oxidized water is equally effective in debris and smear layer removal as compared to NaOCl/EDTA (*p* > 0.05).

## 1. Introduction

Failure of endodontic treatment is mostly related to the remaining microorganisms and their products within the root canal system [1]. Attempts to reduce the microbial load below a critical threshold, which can be subsequently controlled by the immune system [2], usually rely on a combination of mechanical root canal preparation and chemical disinfection [3]. Root canal instrumentation aims to remove necrotic pulp tissues, mechanically disrupt the biofilm matrix and facilitate the flow of the irrigant within the entire length of the root canal [4]. However, endodontic treatment of immature permanent teeth may limit the removal of root dentin to a minimum, since the cessation of root development makes the tooth more susceptible to fracture [5]. In these clinical situations, the debridement of the root canal system is mainly achieved by chemical means [6], which may also require special safety precautions due to the open root apex. It has been reported that nearly half of endodontists experience an accidental irrigant extrusion into the periapical tissues at least once, and open root apices, either iatrogenic or anatomic, may impel the occurrence of irrigant extrusion [7]. Even though various irrigation and irrigant activation techniques have been proposed to avoid inadvertent irrigant extrusion [8], some limitations in terms of safety or cleaning effectiveness usually remain [9]. Therefore, the irrigating solution should be selected appropriately in these clinical cases and should exhibit a high efficacy in root canal disinfection and debridement, along with the absence of toxicity towards the periodontal tissues.

Sodium hypochlorite (NaOCl) is generally assumed to be a benchmark for root canal irrigation due to its exceptional antimicrobial activity, tissue-dissolving properties, low cost and wide availability [1]. Traditionally, the use of NaOCl is followed by the use of calcium chelating agents, such as ethylenediaminetetraacetic acid (EDTA), which dissolve the inorganic part of the smear layer and result in a wider range of open dentinal tubules, thus contributing to the superior debridement and disinfection of the root canal system [10]. The combined application of NaOCl and EDTA has been successfully used in root canal treatment for decades and it is uncertain whether more cost-effective irrigating solutions will be ever found [11]. However, cytotoxicity is a well-known shortcoming of NaOCl that may cause the rapid destruction of periodontal tissues and provoke acute pain, swelling and hematoma when extruded beyond the root canal system [12]. The inadvertent extrusion of NaOCl can be extremely deleterious in immature permanent teeth, leading to the compromised viability of stem cells, which typically reside in close proximity to the root apex and guide the root formation process jointly with Hertwig’s epithelial root sheath [5,13]. Moreover, EDTA associated with NaOCl has proved to induce dentin erosion and adversely affect the dentin microstructure by altering the primary ratio of organic and inorganic components [14]. All these deleterious effects, leading to a significant decrease in dentin’s elastic modulus and flexural strength, as well as potential cellular damage, may jeopardize the long-term prognosis of the tooth [5,15]. Therefore, super-oxidized, or electrochemically activated, water has been previously suggested as one of the potential alternatives for the conventional application of NaOCl and EDTA [16].

Super-oxidized water is typically produced via the electrolysis of dilute salt (NaCl), and contains a mixture of chlorine-based oxidants, such as hypochlorous acid (HOCl), which penetrate the lipid bilayer of the cell membrane and kill pathogens through chlorination or oxidation processes [17]. Previous studies have confirmed that electrochemically activated water exhibits broad-spectrum antimicrobial activity, effectively prevents biofilm formation and leads to a considerable reduction in microbial load, with no significant differences to NaOCl or chlorhexidine gluconate (CHX) [18,19]. These disinfection properties have made super-oxidized water widely used in medicine for wound care and scar management [18]. Successful treatment outcomes, obtained in wound healing [20], suggest that electrochemically activated water provides biocompatibility and favorable cellular responses, making this solution a particularly attractive option for root canal treatment or the management of endodontic complications, where a high risk of irrigant extrusion into surrounding periodontal tissues exists. However, more evidence-based data on super-oxidized water cytotoxicity are needed to confirm its safe biological responses at the cellular level.

Previous studies have shown that electrochemically activated water has little or no impact on the organic component of the dentin matrix [11] and causes fewer dentinal erosion as compared to EDTA [21]. However, the efficacy of super-oxidized water in debris and smear layer removal is still a subject of controversy that needs to be addressed [11,21]. Therefore, in the present study we aimed to evaluate both the potential cytotoxicity of super-oxidized water on cultured human gingival fibroblasts and its efficacy in debris and smear layer removal. The null hypothesis tested was that super-oxidized water is a safe and non-toxic irrigating solution, removing debris and the smear layer from root canal walls with no significant differences compared to the conventional application of NaOCl and EDTA.

## 2. Materials and Methods

### 2.1. Assessment of Cytotoxity

#### 2.1.1. Cell Culture Preparation

Human gingival fibroblasts were grown from a connective tissue graft, which was obtained from a healthy patient undergoing a gingivectomy procedure in the premolar region. The grafted tissue (2–3 mm^3^) was transported in Dulbecco’s modified Eagle’s medium (DMEM; Gibco, Grand Island, NY, USA) supplemented with 250 U/mL penicillin, 0.25 mg/mL streptomycin, 0.05 mg/mL gentamycin and 200 U/mL nystatin. The specimen was subsequently washed with a new portion of DMEM and transferred to Iscove’s modified Dulbecco’s medium (IMDM; Gibco, Grand Island, NY, USA), containing 250 U/mL penicillin, 0.25 mg/mL streptomycin and 20% fetal bovine serum (FBS; Gibco, Grand Island, NY, USA). The grafted tissue was minced under sterile conditions, seeded in 96-well plates and incubated at 37 °C in a 5% CO_2_ atmosphere and 95% relative humidity. The IMDM was changed every 48 h for 2 weeks until the confluence of 80% was reached. Afterwards, the cell monolayer was rinsed in phosphate buffered saline (PBS) without calcium and magnesium, dissociated with 0.25% trypsin/EDTA solution, seeded to new 96-well plates and continued to cultivate. The cell culture of 8–10 passages was used for further analysis.

#### 2.1.2. MTT Assay

Human gingival fibroblasts were seeded in 96-well plates at a density of 5 × 10^3^ cells per 100 µL of IMDM. After the incubation period of 24 h, IMDM was replaced with 100 µL of test medium-Sterilox solution (Optident, Ilkley, UK) + IMDM. Sterilox solution was diluted in IMDM at 30%, 40%, 50%, 60% and 70% concentrations. The control group was maintained in IMDM. All specimens were incubated for 1 h, 2 h, 24 h and 48 h. After the specified time periods, the test medium was removed and 10 μL of 3-(4,5-dimethylthiazol-2-yl)-2,5-diphenyltetrazolium bromide (MTT) was added to each well. Cells with MTT were incubated for 4 h at 37 °C in a humidified 5% CO_2_ atmosphere. The MTT was subsequently aspirated and formazan crystals, produced by dehydrogenases in viable cells, were dissolved in the ethanol. The intensity of the colored solution was measured using a Tecan Infinite 200 absorbance microplate reader (Tecan, Männedorf, Switzerland) at a 570 nm wavelength. Cell viability was evaluated proportionally to absorbance. The mean values obtained from the control group were considered 100% cell viability, and values obtained from experimental groups were expressed as percentages of viable cells proportionally to the control group.

#### 2.1.3. Fluorescence and Phase Contrast Microscopy

Human gingival fibroblasts were incubated for 24 h in test mediums and then treated with 1 μL of dual fluorescent staining solution containing 100 μg/mL acridine orange (AO) and 100 μg/mL ethidium bromide (EB). The 10 µL suspensions of stained cells were placed on a clean microscope slide and covered with a coverslip. Apoptotic cells were visualized under an Eclipse TS100-F fluorescent microscope (Nikon, Tokyo, Japan) at ×200 magnification.

Additional microscopy for the direct identification of pathological cellular changes was performed using an Eclipse TS100-F microscope with a phase contrast set at ×100 magnification.

### 2.2. Assessment of Smear Layer and Debris Removal

#### 2.2.1. Specimen Selection and Preparation

A total of 40 human maxillary premolars having one root canal and fully developed root apices were included in this study, under the approval of the local ethics committee (protocol no. EK-2). Teeth were extracted for reasons unrelated to the study and were stored in an isotonic saline solution at room temperature until use.

Standard endodontic access cavities were prepared with a high-speed Endo Access bur (Dentsply Sirona, Ballaiques, Switzerland) under copious water-cooling. The working length (WL) was determined by inserting a size 10 K-file (Dentsply Sirona, Ballaiques, Switzerland) into the root canal until the tip of the instrument reached the apical foramen and was visible under ×10 magnification (OPMI Pico, Carl Zeiss, Oberkochen, Germany). The WL was established 1 mm from the apical foramen. Afterwards, the root apices were covered with a small amount of sticky wax to prevent the overflow of irrigating solution beyond the apical foramen.

Root canal shaping was performed with rotary nickel-titanium instruments ProTaper NEXT (Dentsply Sirona, Ballaiques, Switzerland) at the established WL. Instruments were driven at the rotation speed of 300 rpm and the torque of 1 Ncm in the following sequence: X1 (17/0.04), X2 (25/0.06), X3 (30/0.07), X4 (40/0.06). After the use of each instrument, root canals were repeatedly irrigated with an open-ended 29-G NaviTip needle (Ultradent Products Inc., South Jordan, UT, USA), attached to disposable syringes. The type of irrigating solution varied between two randomly allocated (www.random.org (accessed on 13 July 2022)) experimental groups (20 teeth per group):

- Sterilox group—root canals were repeatedly irrigated with 2 mL Sterilox solution, containing 200 ppm of available free chlorine. At the end of instrumentation, the irrigation needle was placed 1 mm from the WL and a final 1 min rinse with 4 mL Sterilox, followed by 4 mL distilled water, was performed, moving the needle along the root canal in a 5 mm amplitude.
- NaOCl/EDTA group—root canals were irrigated with 2 mL 3% NaOCl solution (Ultradent Products Inc., South Jordan, UT, USA) after every change of the instrument. The final 1 min flush was performed in the following sequence: 2 mL 3% NaOCl, 2 mL 18% EDTA (Ultradent Products Inc., South Jordan, UT, USA) and 4 mL distilled water.

At the end of the irrigation process, all root canals were dried with sterile paper points. Teeth were prepared by the same operator—an experienced endodontist.

#### 2.2.2. Scanning Electron Microscopy

All specimens were decoronated at the cemento-enamel junction with a diamond-coated high-speed fissure bur and then grooved longitudinally on the buccal and lingual surfaces without penetrating the root canal. Roots were gently split into two halves using a small chisel, and completely dehydrated in a graded ethanol series at room temperature. The examination of each root half was performed under a Stereoscan 100 scanning electron microscope (Cambridge Instrument CO, Cambridge, UK). Ten microscopic fields at ×200 magnification (for debris) and fifteen at ×1000 (for smear layer) were assessed in the apical, middle and coronal thirds. The grading system was used to score the amount of superficial debris as follows:

- Score 1—clean root canal wall, only few small particles of debris;
- Score 2—small agglomerations of debris;
- Score 3—debris covering < 50% of the surface;
- Score 4—debris covering > 50% of the surface;
- Score 5—complete or nearly complete coverage by debris.

The presence of the smear layer was graded as follows:

- Score 1—no smear layer, dentinal tubules are open;
- Score 2—small amount of smear layer, some dentinal tubules are open;
- Score 3—homogenous smear layer covering the surface, only a few dentinal tubules are open;
- Score 4—complete coverage by homogenous smear layer, no open dentinal tubules;
- Score 5—complete coverage by a heavy, non-homogenous smear layer.

The scoring procedure was performed blindly by two trained and independent examiners, who scored each microscopic field from 1 to 5. The mean values of the debris and smear layer were calculated for each root canal third.

### 2.3. Statistical Analysis

Statistical analysis was performed using RStudio software (RStudio Inc., Boston, MA, USA). The assumption of the normality of cell viability data was assessed with the Shapiro–Wilk test and then the homogeneity of variance was confirmed via Levene’s test. Statistically significant differences between data sets were determined using Student’s *t*-test and one-way analysis of variance (ANOVA).

The smear layer and debris scores revealed a non-normal distribution, according to the Shapiro–Wilk test. Therefore, a non-parametric Mann–Whitney test was used for inter-group comparisons, and the Friedman test, followed by the Wilcoxon test, was selected for intra-group comparisons.

The significance level for all comparisons was set at 5%.

## 3. Results

### 3.1. Cytotoxity

**Figure 1:**
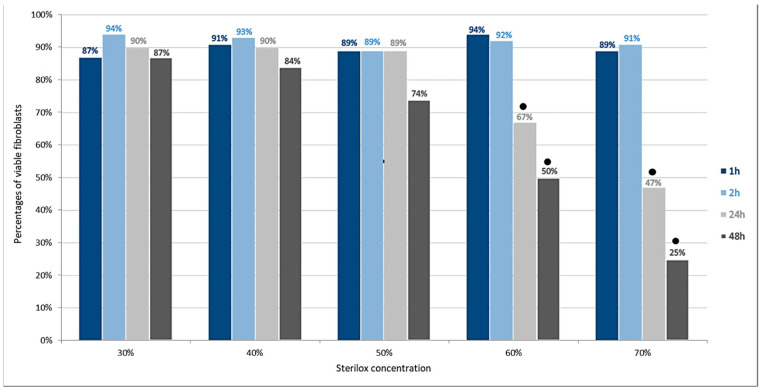
Percentages of viable human gingival fibroblasts after exposure to Sterilox. The black dots (●) above columns indicate statistically significant differences between Sterilox and control groups (*p* < 0.05).

The MTT assay revealed a tendency of Sterilox solutions to reduce cell viability in a concentration- and time-dependent manner. The lowest percentages of viable fibroblasts were observed after 24 h and 48 h incubation at a 70% concentration (Figure 1). No statistically significant differences were detected when the Sterilox and control groups were compared up to 50% concentrations. However, Sterilox was considerably more cytotoxic at concentrations above 50% after 24 h and 48 h (*p* < 0.05).

**Figure 2:**
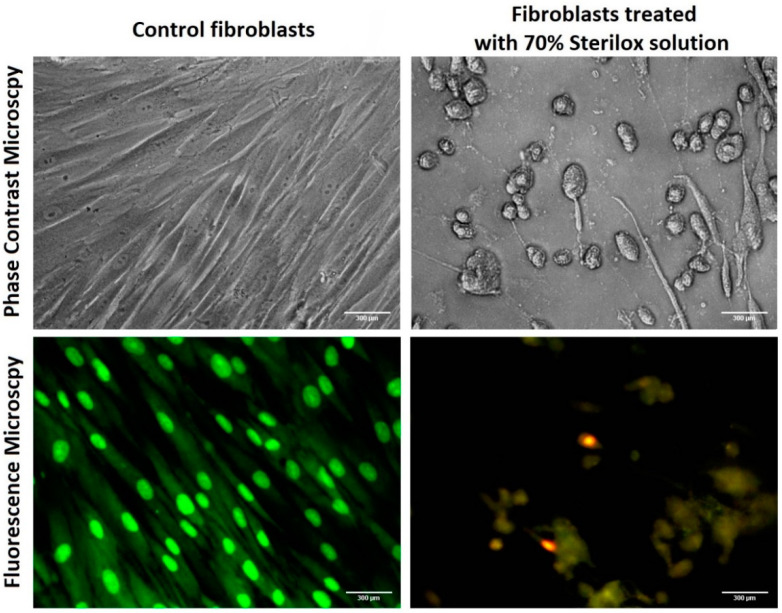
Representative microscopic images of human gingival fibroblasts at ×200 magnification after a 24 h incubation period. The bright red fluorescent coloration indicates the presence of apoptotic cells, whereas healthy viable cells appear green. Scale bar: 300 µm.

The analysis of cell viability was supported by images obtained via fluorescence and phase contrast microscopy (Figure 2). Control cells demonstrated the typical fibroblast morphology, forming a sound monolayer, whereas exposure to Sterilox apparently induced a variety of pathological cellular changes. Apoptotic features at higher Sterilox concentrations appeared as cytoplasmic vacuolization, nuclear shrinkage (pyknosis) and cell rounding.

### 3.2. Smear Layer and Debris Removal

**Figure 3:**
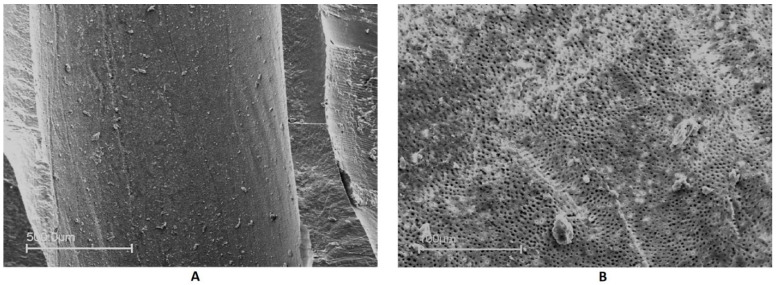
Representative SEM images of (**A**) debris-free root canal surface (middle third) at ×200 magnification; (**B**) smear layer-free root canal surface (coronal third) at ×1000 magnification.

The mean scores of the debris and smear layer are summarized in Table 1. Even though some microscopic fields were free of a smear layer and debris (Figure 3), none of the irrigating solutions used in the present study was able to provide complete cleanliness along the entire length of the root canal.

The amount of smear layer and debris increased from the coronal to the apical third in both experimental groups. However, no statistically significant differences were detected between the Sterilox and NaOCl/EDTA groups (*p* > 0.05) regardless of the lower mean scores observed in the NaOCl/EDTA.

## 4. Discussion

The replacement of NaOCl and EDTA with a non-toxic and less erosive irrigating solution has been regarded as one of the preventive measures to avoid potential damage to periapical tissues and the dentin microstructure [22]. However, a preferred alternative to NaOCl and EDTA has not been identified to date. Even though CHX is usually suggested as an appropriate choice, it has been reported that CHX has little or no tissue-dissolving properties and its concentration recommended for endodontic treatment provides the cytotoxicity similar to that of NaOCl [7]. Moreover, if NaOCl is used to irrigate the root canal, the addition of CHX may lead to brown cytotoxic precipitate formation, with consequent tooth discoloration and a possible negative effect on the sealing ability of theobturation material [23]. Therefore, in the present study we focused on super-oxidized water, which has been previously suggested as another possible endodontic irrigant [24], belonging to the same group of chlorine-based solutions as NaOCl and CHX.

The main active compound of super-oxidized water is hypochlorous acid (HOCl), which dissociates to hypochlorite ions (OCl^−^) only in alkaline environments and has extensively-studied antimicrobial activity against bacteria, fungi and viruses [18,25]. In contrast with hypochlorite ions (OCl^−^), which dominate in NaOCl solution due to its high pH, the HOCl molecule is electrically neutral and can easily penetrate the target cell to exert a strong and rapid bactericidal response [17]. The detrimental effect of HOCl, triggering a variety of cell death mechanisms, has also been detected on different human cell lines [26]. However, evidence suggests that HOCl exhibits its cytotoxicity on human cells in a concentration-dependent manner [27], and only high local concentrations are associated with HOCl-induced cellular damage and oxidative stress [26]. These observations are in accordance with the present study, in which the apoptotic features of super-oxidized water, having an oxidation reduction potential of more than 950 mV, were directly related to the concentration.

The concentration with which HOCl induces irreversible damage to cells may also depend on the cell type [26]. However, the cytotoxicity of HOCl on cultured human gingival fibroblasts has not been investigated previously and thus the mechanism associated with fibroblast resistance to HOCl-mediated oxidative stress remains unclear. It is known that HOCl is a naturally occurring molecule produced by the immune system to destroy invading microorganisms [18]. During the activation of neutrophils, the respiratory burst generates hydrogen peroxide (H_2_O_2_) and subsequently the released enzyme myeloperoxidase catalyzes the formation of HOCl in the presence of H_2_O_2_ [28]. Therefore, it can be speculated that fibroblasts, which play an important role in the process of local inflammation [29], presumably possess a specific defense mechanism providing tolerance to a certain level of HOCl. This has been partially confirmed in a previous study, demonstrating that human gingival fibroblasts at the early stage of inflammation may increase the expression of special enzymes, which repair DNA damage caused by oxidative stress and provide an anti-apoptotic effect for up to 48 h [30]. This short-term self-recovery mechanism may explain the results of the present study, in which the viability of human gingival fibroblasts diminished in a time-dependent manner and reached its lowest level after the incubation period of 48 h. However, a significant decrease in cell viability was observed only with highly concentrated super-oxidized water, whereas concentrations up to 50% successfully retained acceptable biological properties within the specified time periods. These findings partially confirm the null hypothesis associated with the biocompatibility of super-oxidized water and may serve as a guideline for further clinical investigations, which are necessary to confirm the range of clinically safe concentrations, as various cellular processes that are unreproducible by in vitro models may also influence the quantitative extension of HOCl-induced cytotoxicity.

The ability to remove debris and the smear layer from root canal walls is another crucial property for irrigating solutions. Even though it is usually not considered to be of primary importance, non-removed accumulated hard tissue remnants may conceal microorganisms and act as a barrier, preventing the penetration of the irrigant and subsequently the sealer into the dentinal tubules [31]. The results of the present study demonstrated that super-oxidized water has no significant differences in debris and smear layer removal as compared to the conventional root canal irrigation protocol using NaOCl and EDTA. The exact mechanism by which super-oxidized water dissolves tissues is not fully understood. Evidence suggests that the tissue-dissolving properties of chlorine-based solutions are mainly determined by OCl^−^ ions, which begin to be released in low amounts from HOCl at pH values above 5.5 and become the completely dominant form at a pH of 9 [25,32]. However, the OCl^−^ concentration in super-oxidized water is generally assumed to remain low due to its pH range of 5 to 6.5 [24]. Theoretically, these pH values could be increased by the buffering effect of dentin, leading to the higher amount of OCl^−^ ions and thus the more efficient removal of debris and the smear layer [32,33]. Nevertheless, the influence of the dentin-buffering effect on super-oxidized water pH levels has not been investigated to date, and there is also a possibility that a low concentration of OCl^−^ might be compensated for by various other reactive ions and compounds generated during the electrolysis process.

Some limitations of SEM analysis should be also highlighted as one of the factors influencing the results of the present study. Even though SEM is a widely accepted method to assess the ability of endodontic irrigants to remove debris and smear layers [34], this method requires sample sectioning and allows the evaluation only of small selected areas, hence resulting in the potential loss of some valuable information [35]. In order to minimize these limitations, each third of gently halved roots was fully screened under SEM and only areas with the greatest amount of debris and smear layers were selected for further analysis. This evaluation method demonstrated that none of the irrigating solutions used in the present study was able to completely debride the root canal walls, even though some areas were proved to be free of smear layers and debris. The highest amount of residual debris and smear layer was observed in the apical root canal third for both experimental groups. These outcomes might be explained by the limited replenishment and exchange of the irrigating solution in the apical area, despite the sufficient root canal enlargement in the full working length [36]. Previous studies have demonstrated that the mechanical effect of the irrigation process has also a crucial role in the efficacy of root canal debridement [3], and thus various irrigation protocols following sonic or ultrasonic activation of the irrigating solution are highly recommended in terms of debris and smear layer removal [9]. However, the irrigation process in the present study was performed using syringes alone, with no variation in the volume and flow rate of the irrigants, with the aim of assessing the chemical debridement of root canals.

The current findings suggest that super-oxidized water, due to its relative non-toxicity and favorable efficacy in debris and smear layer removal, might be a noteworthy alternative to the conventional application of NaOCl and EDTA. It is not likely that super-oxidized water will ever replace NaOCl and EDTA in daily clinical practice, as it exerts no pulp tissue-dissolving properties [9], retains stability only for 14 days under ideal conditions [11] and contains a lower concentration of available free chlorine than NaOCl, thus requiring a larger amount of the irrigant for adequate disinfection [37]. However, in clinical situations, where irrigant extrusion is a great concern, super-oxidized water could be regarded as a safe and effective option. Further studies, especially well-designed clinical trials, would be highly valuable in order to confirm this hypothesis, as it is impossible to fully reproduce all the clinical conditions and possible biological reactions by means of simplified in vitro models.

## 5. Conclusions

Within the limitations of the present study, it can be concluded that super-oxidized water exhibits an acceptable biocompatibility at concentrations up to 50%, whereas higher concentrations exert apoptotic effects in a time-dependent manner. The efficacy of super-oxidized water in debris and smear layer removal exhibited no significant differences compared to the conventional irrigation protocol of NaOCl/EDTA, even though the superior debridement of root canals was observed in the NaOCl/EDTA group.

## Figures and Tables

**Table 1:** Mean scores and standard deviations of the debris and smear layer for each group in the different root canal third.

Group	Debris (*n* = 200)			Smear Layer (*n* = 300)		
	Coronal Third	Middle Third	Apical Third	Coronal Third	Middle Third	Apical Third
Sterilox	1.42 ± 0.42 ^A^	1.99 ± 0.84	2.17 ± 1.06 ^A^	2.55 ± 1.19 ^B^	3.36 ± 1.22	3.67 ± 0.84 ^B^
NaOCl/EDTA	1.41 ± 0.24 ^C^	1.67 ± 0.72	1.83 ± 0.54 ^C^	2.08 ± 1.11 ^D^	2.53 ± 1.22	2.75 ± 0.93 ^D^

*n* refers to the number of SEM photographs obtained and scored per group in each third. The same superscript letter indicates a significant difference between root canal thirds (*p* < 0.05).

## Data Availability

Data are contained within the article.
